# Can Small Nucleolar RNAs Contribute to Neuropsychiatric Disorders? Insights and Future Perspectives

**DOI:** 10.1016/j.bpsgos.2025.100447

**Published:** 2025-03-14

**Authors:** Yogesh Dwivedi

**Affiliations:** Department of Psychiatry and Behavioral Neurobiology, University of Alabama at Birmingham, Birmingham, Alabama

Small nucleolar RNAs (snoRNAs) are increasingly being recognized as vital regulators of cellular processes, extending beyond their traditional role in ribosomal RNA (rRNA) modification (1). They are of intermediate size (60–300 nucleotides long) and are found across all eukaryotic genomes. It is interesting to note that their numbers rise with the complexity of the organism. For example, while yeast encodes 76 snoRNAs from 64 transcription units, the human genome is predicted to have approximately 2000 snoRNAs. Of these, 505 have been validated in various tissue types such as liver, skeletal muscle, testis, and brain. Even with their widespread presence, not all snoRNAs are actively expressed. Reports indicate that only 13% to 45% of vertebrate snoRNAs participate in active transcriptional processes within the eukaryotic system. This underscores the complexity of their transcriptional regulation, which is highly dependent on their genomic context. Promoter activity and spatiotemporal regulatory mechanisms significantly influence this transcriptional diversity. It has been noted that many snoRNAs are positioned in genomic regions that lack active transcription and essential genomic features such as promoters or necessary flanking sequences, which limits their expression. Most snoRNAs are located within the introns of protein-coding host genes, relying on host gene expression for their production, with only a small subset being transcribed independently. Tissue-specific enrichment in the brain highlights their potential involvement in higher-order processes such as neuronal activity, synaptic plasticity, and cognition.

SEE CORRESPONDING ARTICLE NO. 100415

The canonical role of snoRNAs involves guiding rRNA chemical modifications, including pseudouridylation and 2′-*O*-methylation, which are essential for the proper assembly and function of ribosomes. In this context, our group has recently published a comprehensive review on 2′-*O*-methylation and the role of snoRNAs (2). rRNA modifications are mediated through snoRNA-ribonucleoprotein complexes. snoRNAs are broadly classified into 2 major categories based on their structural motifs and functions: 1) box C/D snoRNAs (SNORDs) and 2) box H/ACA snoRNAs (SNORAs). It is now known that the class of SNORDs direct 2′-*O*-methylation and SNORAs are uniquely positioned to guide pseudouridylation. Due to stoichiometry, these modifications are critical for ribosomal stability and function under complex cellular dynamics. At the same time, it also highlights the indispensable roles of snoRNAs in cellular protein synthesis. However, emerging evidence on tissue-specific snoRNA expression suggests their potential roles beyond ribosome biogenesis. While it is typical for most snoRNAs to exhibit a consistent expression level across tissues, approximately 18% display enrichment in specific tissues, with about 10% predominantly expressed in the brain. This suggests a functional specialization that could influence neural processes.

Comparative studies have revealed significant evolutionary divergence in snoRNA expression among various species. For example, SNORA29 is highly expressed in mice, macaques, and chimpanzees but shows a marked reduction in humans, potentially due to structural instability (3). Although the exact implications of this divergence remain unclear, it raises intriguing questions about the contributions of snoRNAs to species-specific traits, particularly brain development and function. It is increasingly being recognized that in addition to their established role in rRNA processing, snoRNAs may play a significant role in messenger RNA regulation and protein interaction. Certain snoRNAs, such as SNORD115 and SNORD27, have been shown to regulate alternative splicing of critical genes such as serotonin receptor *Htr2c* and the transcription factor E2F7. However, other SNORDs (e.g., SNORD83B and SNORD50A) may contribute to the stability and processing of messenger RNAs. snoRNAs also interact with proteins to regulate key cellular pathways. For example, SNORA73 binds to PARP-1, a nuclear enzyme involved in DNA repair, thereby enhancing ribosome production and cell proliferation (4). These findings collectively illustrate that snoRNAs not only play a regulatory role in ribosomal functions but also serve as active participants in broader regulatory networks.

The new study by Salles *et al.* (5) in *Biological Psychiatry: Global Open Science* provides a comprehensive review of snoRNAs, elucidating the current research trajectory and recent findings that address the missing nuances in the gene regulatory biology of neuropsychiatric and neurodevelopmental disorders. The review also emphasizes the clinical significance of snoRNAs, especially in psychiatric and neurological disorders, where their relevance has become more evident. For example, the review shows that alterations in snoRNA expression are linked to autism spectrum disorder, schizophrenia (SCZ), and major depressive disorder. It is interesting to note that in autism spectrum disorder, dysregulated snoRNA expression in both blood and brain tissues correlated with changes in synaptic gene functions. Alternative splicing was found to be the underlying cause of snoRNA expression changes, which suggests the direct involvement of snoRNAs in causing abnormal neural development. Similarly, SNORD115 has been found to regulate the alternative splicing of the serotonin receptor gene in SCZ brain, most likely affecting both dopaminergic and serotonergic pathways (6). In an earlier report, we showed a significant downregulation of SNORD85 expression together with a significant loss of other small RNAs in synaptosomes derived from the brains of individuals with SCZ; however, other snoRNA-derived small RNAs did not exhibit significant depletion (7). In major depressive disorder, increased expression of SNORA69 has been linked to changes in rRNA modifications, which could possibly lead to the impairment in translation and protein synthesis mechanisms in this disorder (8). These findings point to the critical involvement of snoRNAs in psychiatric disorders and their impact on underlying neurobiological processes. Overall, the complex interactions among snoRNAs, messenger RNAs, and proteins emphasize their roles in broader cellular processes that extend beyond their traditional role as rRNA regulators.

Recent studies have shown the potential of snoRNAs as biomarkers for disease pathophysiology. Their stability in biofluids, such as blood and saliva, makes them attractive candidates for noninvasive diagnostic markers. Interestingly, snoRNAs have been identified in extracellular vesicles, including exosomes, secreted by brain tissues, which serve as cargo for RNA and protein. Due to advancements in sequencing technologies, particularly long-read sequencing, it is now feasible to detect snoRNAs in extracellular vesicles. For example, exosomal SNORD115 and SNORD116 have been shown to serve as biomarkers for Alzheimer’s disease (9). Additionally, snoRNAs may serve as druggable targets with the promise of developing therapeutics to treat abnormalities related to protein misfolding and degradation. In the latest research, snoRNAs such as SNORD90 have been identified as markers associated with antidepressant response (10), highlighting their contribution to therapeutic outcomes. Moreover, modulating snoRNA expression has been shown to influence neurotransmission and behavioral phenotypes in animal models (10), paving the way for novel interventions in psychiatric and neurological disorders.

**Figure 1:**
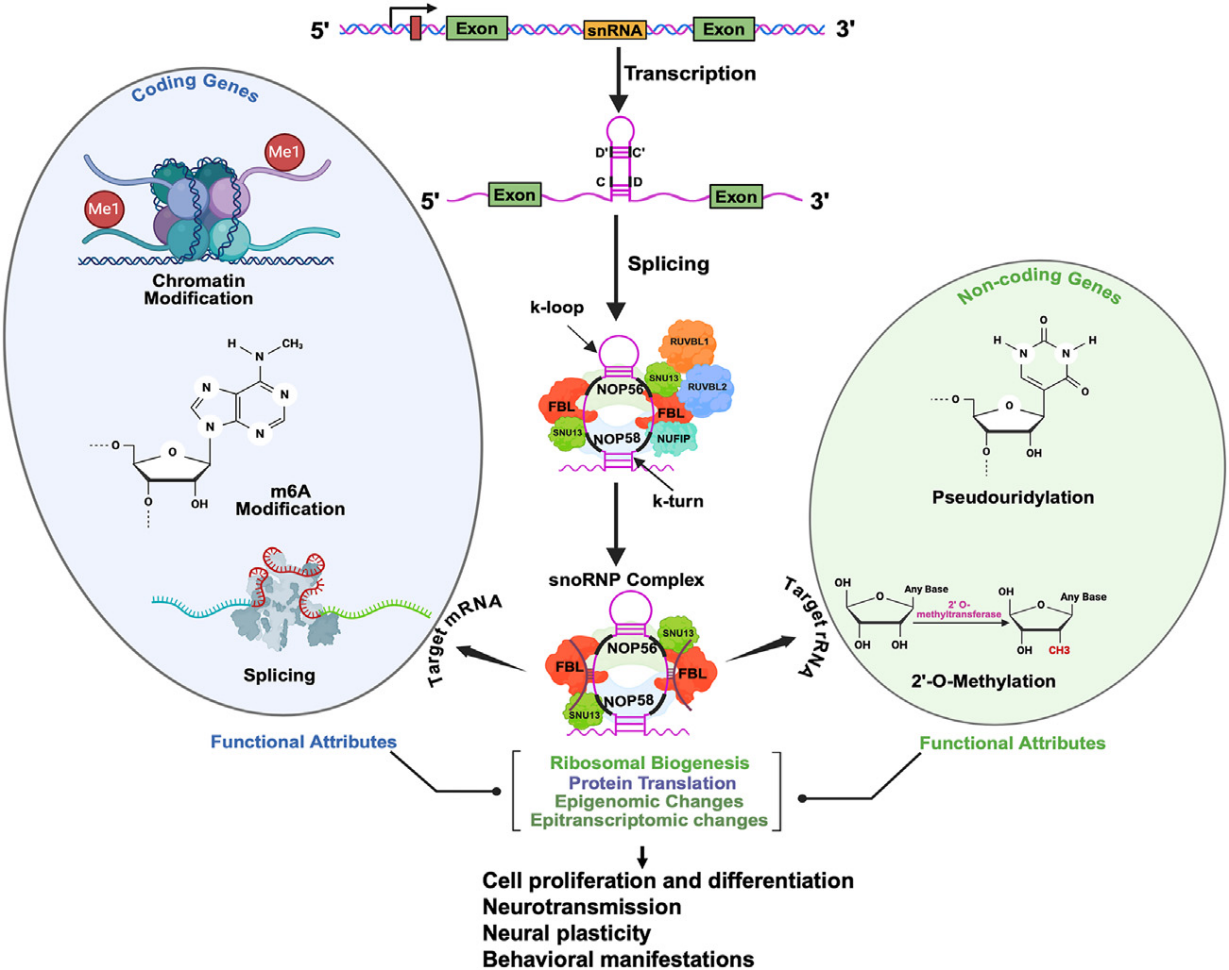
snoRNAs as regulators of RNA functionality and behavior. The figure displays the step-by-step process of snoRNA biosynthesis, which is essential for their maturation and biological roles. It begins with the splicing of precursor snoRNA, followed by exonucleolytic trimming to refine the snoRNA structure. Proteins associated with the C/D box snoRNP complex, such as FBL, NOP56, NOP58, SNU13, NUFIP, and RUVBL1, shield snoRNAs from degradation and assist in their proper folding into functional k-turn and k-loop motifs. The structural conspicuities further assist the snoRNAs in downstream processes such as ribosomal biogenesis and mRNA regulation. The figure also illustrates the diverse functions of snoRNAs in cellular mechanisms, particularly their contributions to ribosome biogenesis and mRNA regulation. Ribosome biogenesis involves the processing of ribosomal RNA through modifications such as 2′-*O*-methylation and pseudouridylation. In mRNA regulation, snoRNAs operate via snoRNPs to influence alternative splicing and epigenomic and epitranscriptomic modifications, such as histone code alteration and m6A marking. The final outcome varies from chromatin structure modulation to protein translation stability. Together, these functions affect cell proliferation and differentiation, cellular signaling, neurotransmission, synaptic plasticity, and neural circuitry, ultimately influencing brain functions and behavior. Figure was created using BioRender.com. k-loop, kink-loop; k-turn, kink-turn; mRNA, messenger RNA; snoRNA, small nucleolar RNA; snoRNP, snoRNA-protein complex.

Despite these advancements, many questions about snoRNA biology remain unanswered. For example, mechanistic studies exploring how snoRNAs influence splicing, methylation, and RNA editing are still in the early stages, particularly in the context of psychiatric disorders. Most research reported to date has focused on descriptive aspects, highlighting correlations between snoRNA expression and disease states without exploring causative mechanisms. As Salles *et al.* (5) rightly pointed out, significant gaps remain despite the recent progress in understanding snoRNA functions. Most studies that have been conducted thus far have been descriptive and limited to bulk tissue analyses and have lacked the resolution necessary to examine snoRNA dynamics at the cellular level. It is highly recommended that future research leverage single-cell sequencing technologies to unravel the cell type–specific roles of snoRNAs. Furthermore, understanding the reversible chemical modifications of snoRNAs is essential for uncovering their regulatory roles. In this regard, investigating m6A methylation at the posttranscriptional level could offer deeper insights. Particularly, how these m6A-based epitranscriptomic modifications can affect snoRNA function in pathophysiological conditions can highlight their biological significance. Another dimension of snoRNA function is their evolutionary adaptability. Findings suggest that snoRNAs may play a role in evolutionary innovations, especially in brain development and function. While it may seem niche, expanding research on snoRNAs’ evolutionary conservation and diversification could also illuminate their roles in species-specific adaptations, including the modulation of ribosomal biogenesis, alternative splicing, chromatin modulation, and epitranscriptomic regulation. These processes are essential for the normal functioning of neural circuits. As mentioned earlier, any pathogenicity associated with snoRNAs can lead to the emergence of complex behavioral manifestations, including major depressive disorder, SCZ, and autism spectrum disorder. In this context, Figure 1 provides an overview of the critical functional pathways that are potentially influenced by snoRNAs, as well as the key steps involved in their biosynthesis, complex regulatory mechanisms, and their functional attributes.

In conclusion, snoRNAs have emerged as versatile regulators of gene expression and cellular function, potentially extending well beyond their traditional role in ribosome biogenesis. The review by Salles *et al.* (5) significantly enhances our understanding of snoRNAs, particularly concerning their roles in tissue-specific regulation, potential links to psychiatric disorders, and their significance as disease biomarkers. As technological advancements continue to improve our ability to study these molecules, snoRNAs are poised to become pivotal contributors in RNA biology and promising targets for precision medicine.
